# A Machine Learning Approach to Uncovering Hidden Utilization Patterns of Early Childhood Dental Care Among Medicaid-Insured Children

**DOI:** 10.3389/fpubh.2020.599187

**Published:** 2021-01-18

**Authors:** Jin Peng, Xianlong Zeng, Janice Townsend, Gilbert Liu, Yungui Huang, Simon Lin

**Affiliations:** ^1^Research Information Solutions and Innovation, Abigail Wexner Research Institute, Nationwide Children's Hospital, Columbus, OH, United States; ^2^Division of Pediatric Dentistry, College of Dentistry, The Ohio State University, Columbus, GA, United States; ^3^Department of Dentistry, Nationwide Children's Hospital, Columbus, OH, United States; ^4^Nationwide Children's Hospital, Columbus, OH, United States

**Keywords:** pediatric dentistry, medicaid, public health, healthcare expenditures, health services research, early childhood dental care, early childhood dental caries, medical-dental integration

## Abstract

**Background:** Early childhood dental care (**ECDC**) is a significant public health opportunity since dental caries is largely preventable and a prime target for reducing healthcare expenditures. This study aims to discover underlying patterns in ECDC utilization among Ohio Medicaid-insured children, which have significant implications for public health prevention, innovative service delivery models, and targeted cost-saving interventions.

**Methods:** Using 9 years of longitudinal Medicaid data of 24,223 publicly insured child members of an accountable care organization (**ACO**), Partners for Kids in Ohio, we applied unsupervised machine learning to cluster patients based on their cumulative dental cost curves in early childhood (24–60 months). Clinical validity, analytical validity, and reproducibility were assessed.

**Results:** The clustering revealed five novel subpopulations: (1) early-onset of decay by age (0.5% of the population, as early as 28 months), (2) middle-onset of decay (3.0%, as early as 35 months), (3) late-onset of decay (5.8%, as early as 44 months), (4) regular preventive care (67.7%), and (5) zero utilization (23.0%). Patients with early-onset of decay incurred the highest dental cost [median annual cost (**MAC**) = $9,499, InterQuartile Range (**IQR**): $7,052–$11,216], while patients with regular preventive care incurred the lowest dental cost (MAC = $191, IQR: $99–$336). We also found a plausible correlation of early-onset of decay with complex medical conditions diagnosed at 0–24 months. Almost one-third of patients with early-onset of decay had complex medical conditions diagnosed at 0–24 months. Patients with early-onset of decay also incurred the highest medical cost (MAC = $7,513, IQR: $4,527–$12,546) at 0–24 months.

**Conclusion:** Among Ohio Medicaid-insured children, five subpopulations with distinctive clinical, cost, and utilization patterns were discovered and validated through a data-driven approach. This novel discovery promotes innovative prevention strategies that differentiate Medicaid subpopulations, and allows for the development of cost-effective interventions that target high-risk patients. Furthermore, an integrated medical-dental care delivery model promises to reduce costs further while improving patient outcomes.

## Introduction

Early childhood caries (ECC) has been a significant oral health problem in many countries, especially in socially disadvantaged populations. ECC is defined as the presence of one or more decayed, missing, or filled tooth surfaces in any tooth in a child under 6 (1). ECC can lead to various adverse outcomes, including toothaches, loss of teeth, sleep disturbances, low self-esteem, and poor school performance (2–5). Children with early childhood caries are at increased risk for future caries and subsequent restorative and surgical treatment that increases costs and risk for complications (6, 7). Nevertheless, dental caries may be largely prevented if preventive measures are applied early. Since early preventive dental visits are critical in preventing dental caries, the American Academy of Pediatric Dentistry recommends that all children have their first preventive dental visit and establish a dental home during the first year of life (8, 9).

We define early childhood dental care (ECDC) as all dental services between 24 and 60 months. ECDC can prevent dental caries and reduce the need for restorative and emergency dental care, therefore reducing dental costs among children (10–12). However, few children received ECDC regularly and even fewer among Medicaid-insured children whose social and economic capital is limited (13–15). Only 20% of Medicaid-insured children receive their eligible preventative dental care (15, 16).

Understanding ECDC utilization patterns is the first step in developing future interventions to prevent early childhood caries and reduce dental costs. Prior studies that examined ECDC utilization patterns have relied exclusively on supervised machine learning methods (e.g., logistic regression) where target outcomes are predefined (e.g., preventive dental care use, yes or no) (17–19). While supervised machine learning is popular and useful in examining relationships between predefined variables, they are incapable of uncovering meaningful hidden patterns that cannot be detected using predefined variables. Unsupervised machine learning methods (e.g., cluster analysis) overcome this limitation by finding clusters within the data using some similarity metric. The objective of this study was to characterize using unsupervised machine learning methods the ECDC utilization patterns among a large cohort of Medicaid-insured children in Ohio. Medicaid-insured children are generally considered as a homogeneous population because they primarily come from low-income families. Breaking away from this usual approach, this study is the first to examine potential heterogeneity in ECDC utilization within a population of Medicaid-insured children.

## Materials and Methods

### Data Source

Founded in 1994, Partners for Kids (**PFK**) is among the largest and oldest pediatric accountable care organizations (**ACOs**) in the United States. Through contracts with five private managed care plans, PFK covers most Medicaid enrolled children (~330,000) in 34 counties in central and southeast Ohio. Forty percent of the children live in Franklin Country, the most urban county in the region, with the remainder spread throughout 33 other counties in Ohio, most of which are rural and many of them Appalachian (20).

In this study, 9 years (2009–2017) of PFK data were used for analysis. Eligible patients (6–60 months) were those who had continuous Medicaid enrollment. The continuous enrollment criteria ruled out a lack of insurance coverage as a barrier to dental utilization. PFK data were complete with no missing data in the variables we used for analysis. About 1% of paid amounts were in negative numbers all of which were converted to zero. All medical claims data (including emergency department visits) were included in our study. Pharmacy claims data were excluded.

A dental visit was defined as the use of any dental services during a single day. We used a combination of Current Dental Terminology (**CDT**), Current Procedural Terminology (**CPT**), and International Classification of Diseases (**ICD**) codes to assign each dental visit into one of the following four mutually exclusive categories.

- **Treatment visit with operating room use (T + OR)**
  A treatment visit was identified as the presence of CDT codes D2000-2999 (restorative procedures), D3000-D3999 (root canal procedures), or D7000-D7999 (oral surgery procedures). Operating room use was identified as the presence of CDT codes D9420, D9219, D9220, D9221, D9223; or the presence of CPT codes (41899, 00170, or 0360) in combination with ICD diagnosis codes for dental disease (ICD-9 of 520-529 or ICD-10 of K00-K14 or M26-M27).
- **Treatment visit without operating room use (T − OR)**
  A treatment visit was identified using the same codes in category (I), but no operating room use was identified.
- **Preventive visit**
  Preventive care was identified using CDT codes D1000-D1999.
- **Other types of dental visits**
  Visits not falling into any of the above categories were identified as other types of dental visits.

In the category assignment process when more than one service was rendered on a given day, category I was given the highest priority because treatment visits and operating room use are considered the biggest drivers of high dental costs. Category II was given the second-highest priority, followed by categories III and IV. For example, if services in both categories I and III were provided during the same visit, we assigned this dental visit to category I.

This study was approved by Nationwide Children's Hospital's Institutional Review Board.

### Data Analysis

Data analysis was performed using Python version 3.6. Costs in dental claims for children between 24 and 60 months were analyzed. We first conducted the traditional segmentation analysis by calculating per member per year (**PMPY**) dental cost by gender. Two sample *t*-tests were performed to examine the significance of differences in PMPY by gender. A *P* < 0.05 was considered statistically significant. We then conducted a data-driven segmentation through cluster analysis.

A cost curve was defined as the cumulative cost of a patient over time. Assuming that a child had three dental visits by 40 months, which incurred dental cost C_1_ at the first visit, C_2_ at the second visit, and C_3_ at the third visit, the cumulative cost of this patient at 40 months will be C_1_ + C_2_ + C_3_. If this patient had another dental visit at the age of 45 months and incurred dental cost C_4_, the cumulative cost by 45 months will accumulate to C_1_ + C_2_ + C_3_ + C_4_. We adjusted the dental expenses using the Personal Health Care index for Medicaid expenditures calculated by the Centers for Medicare and Medicaid Services (21); all amounts are given in 2017 dollars.

Each patient was represented by a concatenated vector of the cost curves of the following four categories: T + OR visits, T − OR visits, preventive visits, and other types of dental visits. We clustered the patients via k-means clustering with Euclidean distance measure (22). The distance between patients is calculated by finding the square of the distance between patients, where each patient is represented by a fixed-length vector. A useful clustering is defined as having a small average distance within a cluster while having a considerable average distance between clusters. We used the elbow method (23) and intuition to determine the optimal number of clusters. We used the silhouette score and Calinski-Harabasz score to compare the goodness of our clustering to a random clustering (24, 25). We used the Kruskal-Wallis test to examine whether the median and mean annual dental costs significantly differ across clusters.

We used three ways to validate the clusters identified via k-means clustering. First, we conducted a dental chart review of four randomly selected patients (two in the early-onset group, one in the late-onset group, and one in the regular preventive care group). Second, we investigated the medical and dental care characteristics at 6–24 months in patients of each cluster. We utilized the Pediatric Medical Complexity Algorithm (**PMCA**) to identify patients with complex chronic diseases (26). Using ICD codes, PMCA stratifies children into three levels of chronic disease: complex chronic disease, non-complex chronic disease, and without chronic disease. We anticipated seeing non-random patterns of medical complexity if the clusters were clinically valid. For example, patients within the same cluster exhibit similar medical complexity levels, while patients from different clusters have significantly different medical complexity levels. Third, we reproduced the analysis in a subset of eligible patients residing in an urban neighborhood (27). Residents from this urban neighborhood have similar socioeconomic status and access to dental care and resources (e.g., fluoride water), thereby ensuring that these variables do not account for the differences in clusters we observed. We anticipated that our urban analysis would reproduce the broader findings.

## Results

We identified 24,223 eligible patients who had continuous Medicaid enrollment between 6 and 60 months. The traditional segmentation by age and gender suggests some differences in dental costs in subpopulations (Table 1). Using k-means clustering, we identified five novel subgroups (Table 2): (1) early-onset of decay (0.5% of population), (2) middle-onset of decay (3.0%), (3) late-onset of decay (5.8%), (4) regular preventive care (67.7%), and (5) zero utilization (23.0%). Our clustering performed better than random clustering (Table 3) and provided more details than the traditional segmentation. Our clustering yielded a better silhouette score than random clustering (0.796 vs. −0.028). For silhouette score, the best value is 1 and the worst value is −1. Our clustering also yielded a better calinski harabasz score than random clustering (0.477 vs. 0.194). For calinski harabasz score, the higher the score, the better the performance.

**Table 1:** The mean of Per Member Per Year (PMPY) dental cost and the total cost of each demographic subgroup (Total *N* = 24,223).

	**% of the population**	**24–36 months**	**36–48 months**	**48–60 months**	**24–60 months total**
Overall	100%	$124.4	$266.4	$278.9	$669.6
Male	51.0%	$134.3	$275.9	$292.0	$702.2
Female	49.0%	$114.1	$256.4	$265.2	$635.7
*T*-test (*p*-value)	–	0.035	0.082	0.051	0.001

**Table 2:** Median and mean annual dental cost by five subgroups of PFK children who had continuously enrolled in Ohio Medicaid from age 24–60 months (Total *N* = 24,223).

**Subgroup**	***N* (% of total population)**	Median annual dental cost of each group^*^ (Interquartile Range)	Mean annual dental cost of each group^*^	**% of total annual dental cost to the ACO**
Early onset of decay	122 (0.5%)	$9,499.2 ($7,052–$11,216)	$9453.6	8%
Mid onset of decay	731(3%)	$5,240.7 ($4,367–$6,006)	$5360.4	25%
Late onset of decay	1,405 (5.8%)	$2,989.5 ($2,483–$3,781)	$3331.4	30%
Preventive care	16,388 (67.7%)	$190.8 ($99–$336)	$357.6	38%
Zero utilization	5,577 (23%)	$0	$0	0%
Total	24, 223 (100%)	$151.2 ($40 - $360)	$669.7	100%

* *p < 0.01, Kruskal-Wallis test*.

*Early, mid, and late onset of decay was defined based on the age when patients had their first treatment visit with operating room use*.

**Table 3:** Performance comparison of our clustering and random clustering.

	**Silhouette score**	**Calinski harabasz score**
Random clustering	−0.028	0.194
Our clustering	0.796	0.477

**Figure 1:**
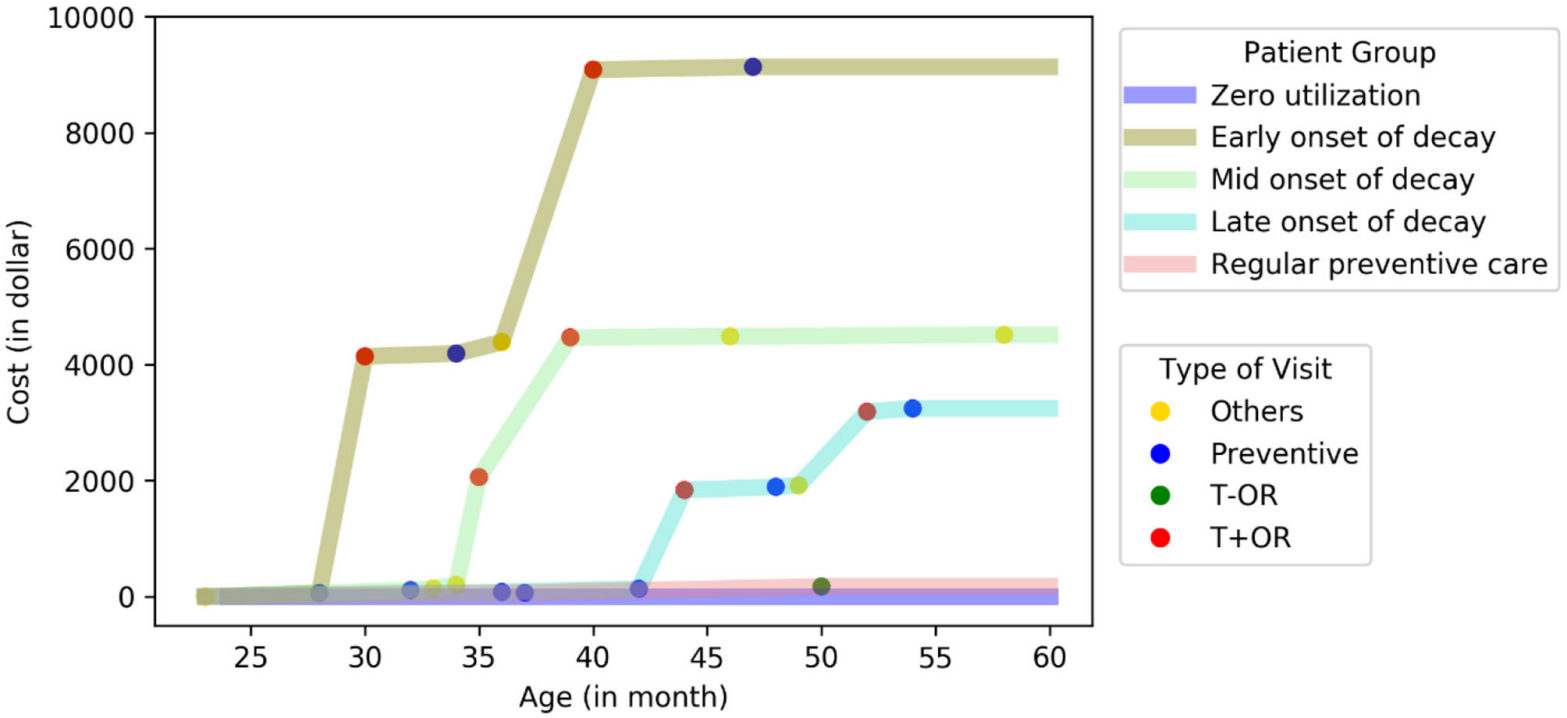
Cost curve of five distinctive subgroups in PFK children who had continuously enrolled in Ohio Medicaid from age 24–60 months. A representative patient at the center of each cluster was plotted (total *N* = 24,223).

We defined subgroups as early-, middle-, and late-onset of decay based on the age when patients had their first treatment visit with operating room use (T+OR). The representative patient from the early onset subgroup had his or her first T+OR visit at 28 months. In comparison, the representative patients from the middle- and late-onset subgroups had their first T+OR visit at 35 and 44 months, respectively (Figure 1). The representative patient from the regular preventive care subgroup had multiple periodic preventive dental visits and never had a T+OR visit from 24 to 60 months. Patients with early-onset of decay incurred the highest dental cost [median annual cost (**MAC**) = $9,499; InterQuartile Range (**IQR**): $7,052–$11,216], while patients with regular preventive care and those with zero utilization incurred the lowest dental costs (MAC = $191 and $0, respectively). Patients in the early-onset group incurred significantly higher dental costs than other subgroups (*p* < 0.01) (Table 2). Noticeably, the patients of early-, mid- and late-onset of decay only constituted 9.3% of the population but consumed 63% of the total annual dental cost to the ACO.

To validate the subgroups, we conducted a dental chart review of four randomly selected charts. The two patients in the early-onset group required dental rehabilitation under general anesthesia soon after their first dental visit indicating that early childhood caries was present. These patients required further dental treatment after the general anesthesia visit, typical with early childhood caries. In the late-onset group, the patient had dental decay diagnosed at 53 months, also requiring general anesthesia. No subsequent dental treatment was needed after the general anesthesia visit, and costs were lower than the early-onset group. In the regular preventive care group, the patient had multiple preventive dental visits but no restorative treatment visits.

As another approach to validate the subgroups, we investigated the resulting clusters' medical and dental care characteristics before 24 months. We observed non-random patterns of the five subgroups before 24 months, which further validated the five subgroups through cluster analysis (Table 4). Noticeably, the percent of patients with complex chronic disease (e.g., congenital heart disease) dropped from 31.2% (early-onset group) to 13.6% (zero-utilization group). The early-onset group had the highest medical cost (median = $7,513) before 24 months, while the zero-utilization group had the lowest medical cost (median = $5,744). The early-onset group came to their first dental visit at the oldest age (median age = 20 months), followed by the mid-onset group.

**Table 4:** Medical and dental care characteristics of the five subgroups prior to 24 months old (Total *N* = 24,223).

	**Medical**			**Dental**	
**Subgroup**	**Median cost of medical visits (Interquartile Range)**	**Median number of medical visits (Interquartile Range)**	**% classified as medically complex patients**	**% had a dental visit**	**Median age of first dental visit in months (Interquartile range)**
Early onset of decay	$ 7,513 ($4,527–$12,546)	15 (10–28)	31.2%	29.5%	20 (14–19)
Mid onset of decay	$ 6,550 ($3,993–$11,210)	15.5 (9–24)	20.4%	30.5%	18 (10–20)
Late onset of decay	$ 6,399 ($3,957–$10,763)	16 (10–25)	21.8%	24.7%	16 (12–20)
Preventive care	$ 6,491 ($4,003–$10,824)	16 (10–25)	17.5%	22.3%	16 (12–20)
Zero utilization	$ 5,744 ($3,545–$9,710)	17 (11–27)	13.6%	10.4%	14 (12–20)
Overall	$ 6,300 ($3,892–$10,588)	16 (10–25)	17.0%	20.0%	16 (12–20)

*Early, mid, and late onset of decay was defined based on the age when patients had their first treatment visit with operating room use*.

*Silhouette score: The best value is 1 and the worst value is −1*.

Calinski harabasz score: The higher the score, the better the performance.

**Figure 2:**
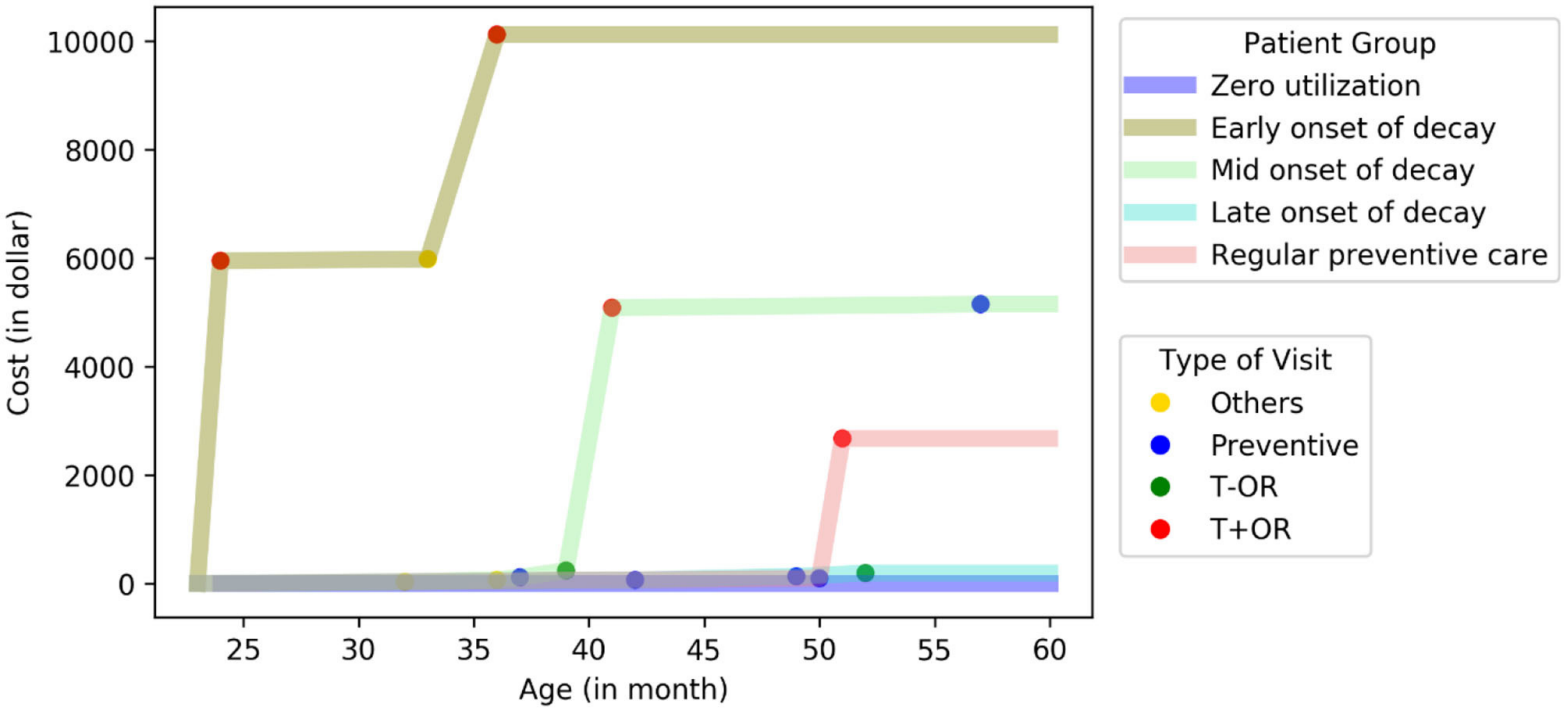
Reproducibility of the five subgroups in an urban subpopulation. A representative patient at the center of each cluster was plotted. (total *N* = 2,250).

To further validate the subgroups, we reproduced the analysis in an urban subpopulation of eligible children. We observed a similar pattern of five subgroups (Figure 2), which consisted of 0.4% (early-onset), 2.5% (mid-onset), 4.5% (late-onset), 35.5% (preventive), and 57.1% (zero utilization) of the subpopulation, but consumed 12, 38, 39, 11, and 0% of the total dental cost, respectively.

## Discussion

Our study is the first to analyze pediatric dental claims data by calculating cumulative dental cost per person over multiple years (2009–2017). Our study is also the first to use unsupervised machine learning to characterize ECDC utilization among Medicaid-insured children. Using cluster analysis of accumulative cost curves, we identified five subgroups with distinctive clinical, cost, and utilization patterns among Ohio Medicaid-insured children: (1) early-onset of decay, (2) mid-onset of decay, (3) late-onset of decay, (4) regular preventive care, and (5) zero utilization. These subgroups are clinically meaningful and validated through patient chart review and characterization of each subgroup before 24 months. The five subgroups have also been reproduced in an urban subpopulation.

### Comparison to Prior Studies

The five subgroups discovered in this study have not been previously reported. Nevertheless, our other results are comparable to findings from prior studies.

We found that the average cost for early-, mid- and late-onset groups were $9453.6, $5360.4, and $2,989.5 respectively (Table 2). This cost range is consistent with previous reports. In 2000, the average cost to Medicaid for dental treatment under general anesthesia was $2,009 per case in Iowa (28). In 2018, the average cost for dental treatment under general anesthesia in a hospital setting was $9,833.79 (range = $2,062-$16,620) and $1,955.38 (range = $1,250-$3,525) in an office setting excluding professional fees (29). The use of general anesthesia for Medicaid-insured children is increasing and has significantly driven up Medicaid dental expenditures (30). Approximately 0.5% of Medicaid-insured children required general anesthesia at the cost of $68 million in 6 states, which extrapolates to $450 million nationally (31). Therefore, general anesthesia is likely the driving force of high dental costs for our three decay onset groups. Also, the recurrence of dental caries and restoration failure is exceedingly common among children treated under general anesthesia. When less durable restorations (e.g., composite restorations and strip crowns) are applied during general anesthesia, the failure rate is high, suggesting more treatments under general anesthesia (32). These additional treatments are likely another driver of the high cost for the three decay onset groups. Furthermore, atraumatic restorative techniques [e.g., silver diamine fluoride (SDF)] were not a covered benefit in Ohio Medicaid during our study period (2009–2017). This lack of covered benefit may have also contributed to the high dental costs observed in this study because repeated use of general anesthesia could have been potentially prevented if Ohio Medicaid covered SDF (33–35).

We also found that the early-onset group had their first dental visit at the oldest age and incurred the highest dental cost. This finding is consistent with prior studies that reported on ECDC utilization and costs. Nowak et al. found that children who started dental care at younger than 4 years of age had less restorative and surgical treatment than children who began dental care at an older age (11). Savage et al. also found that dental cost increases as the age at first preventive dental visit increases (10).

Furthermore, we found that the early-onset group had the highest percentage of patients with complex medical conditions (31.2%) and the highest medical cost (MAC = $ 7,513) before 24 months. This finding revealed an interplay between medical and dental conditions, which is consistent with previous findings. Craig et al. found that children with special healthcare needs (**SHCN**) had more caries and were less likely to use preventative dental care (19, 36). SHCN has been classified as a moderate risk for dental caries in children at 0–5 years (37). Chi and colleagues found that children with autism were less likely to utilize preventive dental care than those without autism (38). All those findings indicated that children with complex medical conditions tend to have more dental conditions.

### Implications for Public Health Interventions

The early onset group included a small number of children (0.5% of the study population) but incurred disproportionately high costs (8% of the total dental cost to PFK). The early-onset group's representative patient had dental treatment under general anesthesia almost immediately after the first dental visit, indicating the child came to the dentist for the first time with severe dental caries (Figure 1). Caregivers of children in this early-onset group are likely to have low oral health literacy and few resources for finding dental care; and the children are not receiving any preventive dental care. Thus, the early-onset group's interventions should occur within their first 2 years of life and involve proactive outreach to their families. Support from primary care providers is essential but likely inadequate to connect those children with dental homes. An integrated medical-dental care delivery model may be a more viable and efficient approach to connect those children with dental homes. PFK has existing care coordination programs to help patients navigate and adhere to care with multiple healthcare providers. Dental care can be added as a critical component to the PFK care coordination program to proactively connect children with dental homes at an early age through home visits. This integrated care delivery model will further increase patient-centered care management and advocate for vulnerable children in many other ways.

The mid-onset group also included a small number of children (3% of the study population) and incurred disproportionately high costs (25% of the total dental cost to PFK). Compared to the early-onset group, we have a better chance of helping children in this group defer existing caries' progression and prevent subsequent caries. Primary care providers can play an essential role in preventing early childhood caries by incorporating oral health as a vital component of a routine well-child visit. Primary care providers can educate parents on healthy oral-health behaviors, provide fluoride varnish, and refer patients to establish a dental home by age one. Early Head Start programs can also support oral health by including dental screening and anticipatory guidance as a critical component of routine services such as child care (39, 40).

The late-onset group comprised 5.8% of the study population and incurred 30% of the total dental cost to PFK. Caregivers of children in this group likely have some oral health literacy level and necessary resources to access dental care; they may therefore have the potential to be caries free with early preventive interventions. Primary care providers can play an essential role in helping those children by assessing their oral health and related behaviors at well-child visits. Primary care providers can educate parents on healthy oral-health behaviors, place fluoride varnish, and refer patients to a dental home. With the early establishment of a dental home, incipient caries can be treated with chemotherapeutic and minimally invasive restorative treatments which may avoid the need for general anesthesia (37). Early Head Start programs also can connect these families to dental care resources (39, 40).

The preventive care group comprised 68% of the study population. The preventive care group accounted for the largest share of the total annual dental cost to the ACO (38%) (Table 2). However, this group had the lowest associated median ($190.8) and mean ($357.6) annual dental cost among all the groups examined in this study. Future studies should examine this group in more depth to identify protective factors that can be encouraged in other groups.

The zero-utilization group comprised 23% of the study population. This percentage is consistent with previously published data where 28% of Medicaid-insured children in four states did not receive any dental services (41). We know little about the children in the zero-utilization group. These children will be older when they present to the dentist. Due to the lack of early preventive dental care, they are likely to have a high burden of dental disease. Despite the increased maturity, these children may still need general anesthesia as they have not developed the coping skills acquired during routine dental visits. At age 6, their permanent teeth will start erupting, and lack of ECDC may detrimentally impact their permanent dentition leading to a lifetime of dental compromise. The presence of a dental home should be assessed at well-child visits, and a referral made if the children have not seen a dentist. School-based dental programs may identify these children and connect them to dental care resources, but proactive care coordination at an earlier age would be ideal.

### System-Level and Family-Centered Strategies

The costs observed in this study only reveal the tip of the iceberg of early childhood caries' impact on Medicaid-insured children and their families. Loss of a job, loss of income for time spent taking a child to dental appointments, missed school days, travel expenses, and mental and physical stresses are real and significant barriers to these families, exacerbated in today's chaotic economy (29, 42). System-level and family-centered strategies are warranted to mitigate those barriers and improve oral health for Medicaid-insured children.

Under the Early and Periodic Screening, Diagnostic and Treatment (**EPSDT**) benefit (43), state Medicaid programs must provide comprehensive and preventive health care services for Medicaid enrolled children under age 21. This provision includes dental care, regardless of whether such services are covered for adults or included in the state plan. Despite comprehensive coverage through EPSDT, access to dental care remains a barrier. Only 38 percent of dentists participate in Medicaid; low reimbursement rates are one reason cited by dentists for not participating (44). Although fluoride varnish in primary care has been promoted, oral health remains a low priority in Ohio Medicaid. To improve access to dental care among Medicaid enrolled children in Ohio, reimbursement incentives are needed to encourage dental care providers to participate in Ohio Medicaid. To integrate oral health into the overall health care system, national organizations, including the American Academy of Pediatric Dentistry, promote the establishment of a “health home.” This health home would bring together the interaction of the child, parents, non-dental health professionals, and dental professionals to deliver medical and dental care in a coordinated, integrated, and family-centered way (45). Strategies are also needed to provide a sufficient and effective dental workforce and assure health professionals' appropriate training on ECDC management and parent education.

Beyond the healthcare system, oral health should also be coordinated with care systems supporting young children (e.g., childcare centers and schools). Childcare providers, teachers, and school administrators must be engaged as partners to promote early childhood oral health. They must know the origin and associated risk factors for tooth decay, be empowered to make appropriate decisions regarding timely and effective interventions, and facilitate dental care for young children (46, 47).

**Figure 3:**
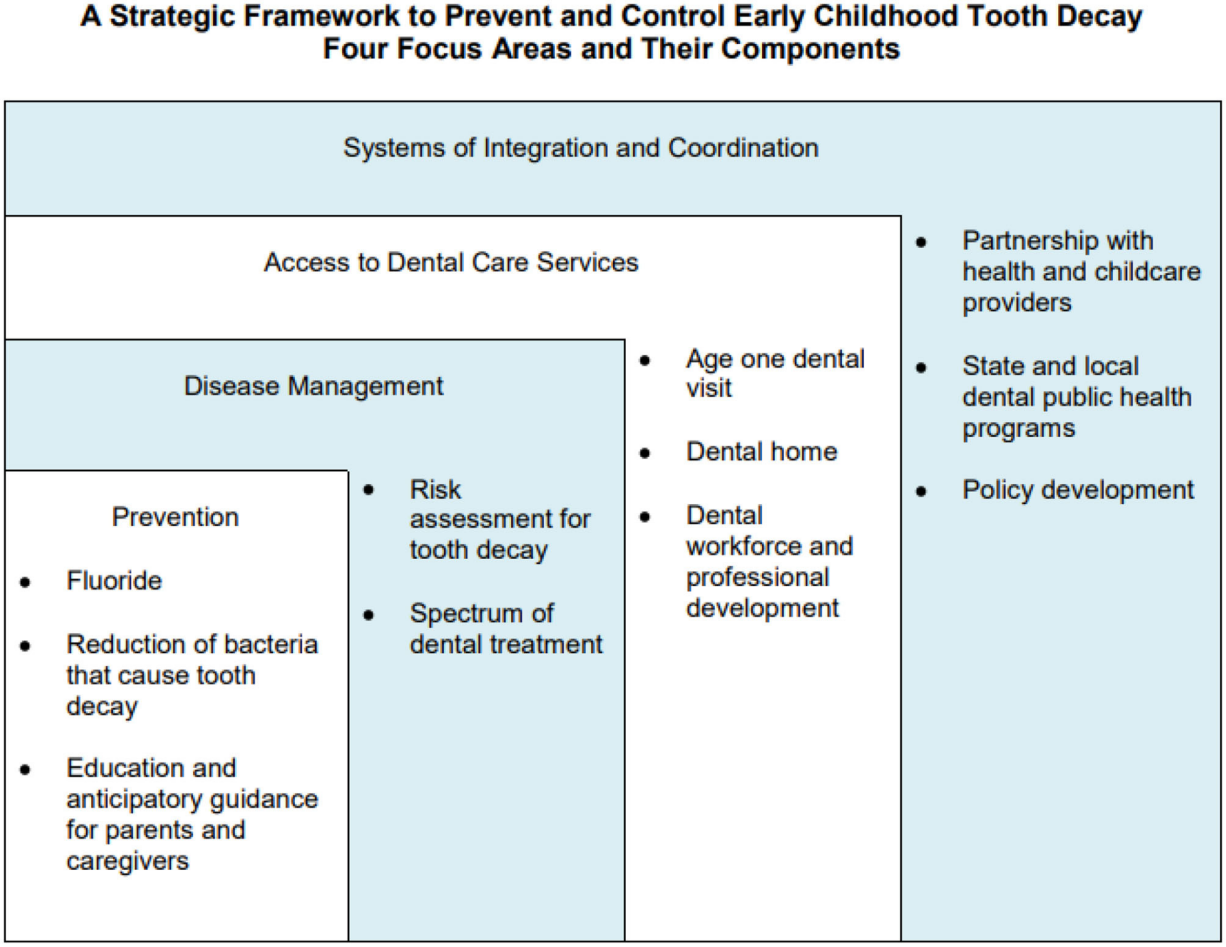
A strategic framework to improve ECDC.

Fisher-Owens and colleagues proposed a model that recognizes the levels of influence on children's oral health and shows that child, family, and community interact with the biological factors impacting oral health (48). Drawing on this model, the Association of State and Territorial Dental Directors developed a strategic framework to prevent and control early childhood caries (45). The framework (Figure 3) includes four focus areas: Prevention, Disease Management, Access to Dental Services, and Systems of Integration and Coordination that are tied to the child, family, and community levels of influence on children's oral health. This framework can help plan and implement strategies, develop policies, conduct research, and allocate resources to prevent early childhood caries and improve early childhood oral health. Local, state, and national efforts should focus on these four areas to strengthen early childhood oral health.

### Limitations and Future Studies

This study had several limitations. First, our findings were based on data from one state and may not be generalizable to other states. Each state operates its own Medicaid program within federal guidelines. Because the federal guidelines are broad, states have a great deal of flexibility in designing and administering their programs. As a result, Medicaid eligibility and benefits often vary widely from state to state. Also, similarities in training and practice patterns among Ohio dental providers may limit our findings' generalizability to other states. Future studies are warranted to assess whether our results can be replicated in other states. Second, we limited our study population to those with continuous Medicaid enrollment from 6 to 60 months of age. Although some potentially high-cost patients may be excluded due to discontinuous enrollment, continuous enrollment criteria have been widely used in the literature to obtain complete longitudinal data to enable sound data analysis (49). Third, no race and ethnicity information were available in our data. Dasanayake et al. examined dental care utilization among Alabama Medicaid-insured children and found significant racial disparities in dental service utilization among those children (50). Future studies should further explore racial disparities in ECDC utilization among Medicaid-insured children. Fourth, although our data revealed an interplay between complex medical and dental conditions, we were not able to explore this topic in-depth in the current study. Frank et al. suggested that the extent of caries varies among different subgroups of children with SHCN (36). Future studies should further explore the relationship between SCHN and early-onset of dental caries. Furthermore, future analysis is warranted to identify geographical barriers to optimal oral health including lack of dental providers, lack of fluoridated water, or lack of access to healthy foods in some geographical areas. Targeted interventions such as mobile dental clinics, increased fluoride varnish applications by medical providers, or access to bottled fluoridated water may be cost-effective in a managed care population. Once we can predictably identify these groups, surveys can be implemented to understand better behavioral risk factors such as sugar intake or oral hygiene habits.

## Data Availability Statement

The data analyzed in this study is subject to the following licenses/restrictions: Researchers must sign a data use agreement to use the dataset. Requests to access these datasets should be directed to https://partnersforkids.org.

## Author Contributions

JP: contributed to data acquisition, study design and results interpretation, and drafted and critically revised the manuscript. XZ: contributed to study design, results interpretation, and performed all data analyses. JT: contributed to results interpretation, performed medical chart review, and drafted and critically revised the manuscript. GL: contributed to results interpretation and critically revised the manuscript. YH: critically revised the manuscript. SL: contributed to conception, study design and results interpretation, and critically revised the manuscript. All authors: gave final approval and agreed to be accountable for all aspects of the work.

## Conflict of Interest

The authors declare that the research was conducted in the absence of any commercial or financial relationships that could be construed as a potential conflict of interest.
